# KICK OUT PD: Feasibility and quality of life in the pilot karate intervention to change kinematic outcomes in Parkinson’s Disease

**DOI:** 10.1371/journal.pone.0237777

**Published:** 2020-09-09

**Authors:** Jori E. Fleisher, Brianna J. Sennott, Erica Myrick, Claire J. Niemet, Monica Lee, Courtney M. Whitelock, Maya Sanghvi, Yuanqing Liu, Bichun Ouyang, Deborah A. Hall, Cynthia L. Comella, Joshua Chodosh

**Affiliations:** 1 Department of Neurological Sciences, Rush University Medical Center, Chicago, Illinois, United States of America; 2 Rush Medical College, Rush University Medical Center, Chicago, Illinois, United States of America; 3 Yale College, Yale University, New Haven, Connecticut, United States of America; 4 Department of Medicine, New York University School of Medicine, New York, New York, United States of America; 5 Medicine Service, VA New York Harbor Healthcare System, New York, New York, United States of America; Universidade Federal do Rio Grande do Sul, BRAZIL

## Abstract

**Background:**

Multiple exercise modalities and mindfulness activities are beneficial in Parkinson’s Disease (PD). Karate is a martial art that combines aerobic and large-amplitude movements, balance and core training, and mindfulness, suggesting a potential benefit for individuals with PD from multiple perspectives.

**Objective:**

To evaluate the feasibility of community-based *Shotokan* karate classes involving physical activity and mindfulness among individuals with mild- to moderate-stage PD, and to explore the effects of karate on objective and patient-reported outcomes.

**Methods:**

We conducted a 10-week, unblinded trial of twice weekly, PD-specific karate classes. Feasibility was assessed by: dropout rates, adherence via attendance records, adverse effects and falls, and continued participation six months post-intervention. Participants completed pre- and post-intervention assessments of disease-related quality of life (Parkinson’s Disease Questionnaire-8, PDQ-8), falls, and post-intervention assessment of change in overall wellbeing (Patient Global Impression of Change, PGIC), with exploratory measures of mobility using the Timed Up and Go (TUG), mood using the Hospital Anxiety and Depression Scale (HADS), and cognition using digit span forward and backward and the Symbol Digit Modalities Test (SDMT).

**Results:**

Of 19 enrolled participants, 15 completed the study (79%). Among completers, mean adherence was 87% during the ten weeks of intervention, and 53% maintained karate participation six months later and endorsed sustained improvement, respectively. No adverse effects or change in fall frequency were detected. Among completers, 53% were women, and mean PD duration was 6 years (range 2–20). Quality of life improved to a clinically significant degree (PDQ-8: mean 25.3 (standard deviation (SD) 20.8) versus 19.3 (SD 19.6), p = 0.01, effect size 0.83). On the PGIC, 87% endorsed feeling moderately or considerably better. Mobility did not change significantly (TUG: 9.6 seconds (SD 2.23) versus 9.0 seconds (SD 1.89), p = 0.12, effect size 0.43), nor were there changes in overall physical activity, mood, or cognition (p = 0.35–0.92).

**Conclusions:**

In a small, 10-week, unblinded trial of community-based karate classes for individuals with mild and moderate PD, high adherence was noted. Quality of life and wellbeing improved significantly, without changes in exploratory outcomes of mobility or neuropsychological outcomes. The study was underpowered, particularly for the exploratory outcomes. Controlled and longitudinal investigation is warranted to confirm our pilot findings and explore the long-term effects and sustainability of karate in PD.

**Trial registration:**

Clinicaltrials.gov: NCT03555695.

## Introduction

Parkinson’s Disease (PD), the second most common neurodegenerative condition, impacts mobility, functional abilities, and quality of life [1]. Exercise is an established supplement to pharmacologic treatment and various modalities have demonstrated benefits in PD. Overall, physical activity has been shown to improve non-motor symptoms [2], as well as specific motor symptoms including gait, balance, and quality of life measurements [3]. Aerobic activity appears to have disease-modifying effects, demonstrating physical improvements and increased quality of life [4,5]. Mindfulness-based movement practices, such as tai chi and qigong are also beneficial for both motor and non-motor manifestations of PD [6,7]. Specifically, tai chi leads to greater improvements in balance and postural stability, and a higher likelihood of continued exercise when compared with traditional resistance training [8].

Karate is a martial art incorporating aerobic and resistance exercise with mindfulness practice similar to tai chi. Karate is taught as large, directed movements in a class-based, communal environment. At the elite competition level, karate can elicit near-maximal exertion as measured through cardiovascular parameters [9], supporting the premise that the large movements, involving multiple muscle groups, can offer physical challenge and exertion to those at the beginner level, participating in community classes. Furthermore, karate has been shown to have benefits when compared with a non-active mindfulness intervention among healthy older adults [10] however, karate as an adjunct therapy for PD has been minimally investigated. A 1986 study demonstrated physical and psychological benefits of both a seated karate program and a traditional, PD-specific exercise program [11]. More recently, a study where participants self-selected into an inactive control group, dance training, or karate training demonstrated improved balance and stable mood in dance and karate, and greater adherence to the karate intervention [12].

Based on the benefits of both exercise and mindfulness in PD, and of karate interventions in non-PD older adult cohorts, karate interventions appear fit for study. Karate may be a beneficial addition to the repertoire of exercises available to those with PD because it incorporates the physical challenge and exertion seen in many exercise modalities, with the mindfulness and breathing usually associated with less strenuous activities, such as tai chi. Additionally, karate may appeal to individuals who are interested in the striking and combat component, which is not represented in tai chi or aerobic exercises, and they would also benefit from the mindfulness focus, which is difficult to find in other popular, combat-adjacent exercises, such as boxing. Therefore, investigating this particular martial art is valuable because it will build our knowledge about the feasibility and potential benefits of another exercise option for individuals with PD.

For this pilot study, we implemented and tested the feasibility of KICK OUT PD (*K**arate* *I**ntervention to* *C**hange* *K**inematic* *OUT**comes in* *PD*), a community-based karate group-training class tailored to individuals with mild and moderate PD (Hoehn & Yahr stages 1–3) [13]. Additionally, we report on PD-specific quality of life, patient global impression of change, adverse events and fall frequency, and self-reported physical activity. As exploratory outcomes, we also assessed mobility, cognition, and mood.

## Materials and methods

### Study design

We conducted a ten-week, unblinded trial of twice-weekly, structured, non-contact karate classes tailored for individuals with mild- to moderate-stage PD following the Transparent Reporting of Evaluations with Nonrandomized Designs (TREND) statement [14]. Our primary objective was to evaluate the feasibility and acceptability of community-based karate classes among individuals with mild- to moderate-stage PD. We also hypothesized that karate may offer positive effects on mobility, balance, and quality of life in this population. The feasibility and acceptability of this exercise intervention were measured via participant withdrawal/dropout, adverse effects and falls, and class attendance. As exploratory secondary outcomes, we assessed quality of life, patient global impression of change, and mobility at pre- and post-intervention study visits. We assessed sustainability via six-month follow-up phone calls regarding continuation of karate practice. The Institutional Review Board at Rush University Medical Center approved this study (Clinicaltrials.gov: NCT03555695). Written informed consent was obtained from each participant.

### Participants

We recruited individuals with PD from the Rush University Parkinson’s Disease and Movement Disorders Program and other Chicago-area institutions between March-June 2018. Recruitment methods included direct outreach to intramural and extramural neurologists, senior centers, and libraries throughout Chicago. We placed recruitment postings in institutional newsletters and fliers in the neurology offices. Within Rush, we queried our Institutional Review Board-approved data repository for a list of English-speaking individuals diagnosed with PD.

Inclusion criteria were as follows, gleaned first from chart review and subsequent telephone screening: PD diagnosed according to UK Parkinson’s Disease Society Brain Bank Clinical Diagnostic Criteria [15]; between 30 and 90 years of age; English-speaking; seen as an outpatient within the last two years; and able to attend classes twice weekly at one of two karate studios located in the Chicago suburbs. Study team members (JF, BS, CN, ML, CW) prescreened and contacted potential participants based on the inclusion criteria above, utilizing the most recent outpatient visit. For those recruited through community outreach, all were screened for eligibility and we required confirmation of PD diagnosis and Hoehn & Yahr stage from the referring neurologist prior to study enrollment. We included participants regardless of treatment status, including pharmacologic or surgical treatment for PD. Participants were encouraged but not required to maintain their treatment regimen for the 10-week study.

Exclusion criteria were as follows: Individuals living >20 miles from participating karate studios; a diagnosis of atypical parkinsonism, including Progressive Supranuclear Palsy, Multiple System Atrophy, Dementia with Lewy Bodies, Corticobasal Syndrome, drug-induced or vascular parkinsonism, or other atypical parkinsonism not otherwise specified; Hoehn and Yahr stage 4 or higher (either directly noted in chart review at the most recent visit, or indicated during the screening phone call by the individual requiring an assistive device to ambulate); severe psychiatric disorders (defined as persistent hallucinations or delusions with lack of insight, or severe anxiety or depression requiring inpatient hospitalization or suicidal ideation in the preceding 30 days); participation in martial arts and/or boxing exercise programs in the 30 days prior to study entry; or inability to commit to attending or traveling to two classes weekly for 10 weeks. Although subjects were encouraged to attend all 20 sessions, we did not impose a minimal attendance exclusion criterion.

Due to the greater availability of instructors and space at the karate studios during the summer, and the availability of medical student study team members (BJS, ML, CMW) to support participants by attending classes, convenience sampling was used to establish the study cohort. The goal was to recruit and enroll all participants in order to conduct the 10 weeks of classes during the summer months, for the reasons above.

### Sample characterization, assessments, and outcomes

Prior to the start of classes, participants attended a pre-intervention group visit at Rush University. At this session, participants completed a series of self-report measures for sample characterization, starting with a brief demographic and history questionnaire covering PD diagnosis, prior involvement with martial arts, and expectations related to karate class. To assess baseline fall frequency, the study team defined a fall as any “unintentional going to the ground” before subjects responded to the following question: “How frequently have you fallen in the last twelve months?”, with response choices of “Never”, “Rarely”, “Monthly”, “Weekly”, or “Daily”. To assess baseline activity level, participants completed the International Physical Activity Questionnaire (IPAQ), a validated questionnaire that asks participants to reflect on the past seven days and indicate the number of days, and number of minutes per day, spent in vigorous physical activity, moderate physical activity, walking, and sitting [16]. The IPAQ defines “vigorous” versus “moderate” activity using examples such as bicycle pace and lifting heavy or light loads.

Feasibility assessments included withdrawal rate from the study, adverse effects and fall frequency during the ten-week study period, attendance, achievement of personal goal, likelihood of continuing, and likelihood of recommending karate to a friend with PD. Attendance records were maintained by karate studio staff at each class and sent to the study team. Attendance was calculated as the percentage of classes attended out of a total of twenty possible classes. The study staff contacted any participants missing >2 classes in a row to assess the reason for missed classes, including any adverse effects or intent to withdraw from the study. Study staff attempted at least 3 phone calls to gather data on missingness or withdrawal. Adverse effects were noted either by study or karate studio staff identifying potential adverse effects during class and following up by phone call with the participant, or elicited during phone calls for missingness or withdrawal. At the pre-intervention visit, participants were asked to write down a specific, personal goal that they hoped to achieve through karate. At the post-intervention visit, participants were presented with and asked to indicate whether or not they achieved the personal goal they set at the pre-intervention visit (yes/no). They were asked to provide a binary responses to whether they would recommend the program to someone else with PD, and whether they would continue participation in karate classes, if available.

Secondary outcomes included quality of life and global impression of change. We assessed quality of life using the Parkinson’s Disease Questionnaire-8 (PDQ-8), a brief, validated, PD-specific measure of quality of life. The PDQ-8 yields a summary index score range of 0–100, where higher scores signify worse quality of life [17]. To assess change in overall wellbeing, we administered the Patient Global Impression of Change (PGIC) [18]. The PGIC assesses the individual’s subjective, overall response to an intervention using a seven-point rating scale, ranging from “No change, or worse” (1), “A little better, but no noticeable change” (3), “Moderately better, and a slight but noticeable change” (5), to “A great deal better and a considerable improvement that has made all the difference” (7). Scores of 5–7 are considered clinically meaningful improvements [19,20]. As the PGIC’s single item refers to a change from baseline, it was only administered at the post-intervention visit and six-month follow-up phone call.

Exploratory outcomes included mobility, balance, gait, and flexibility. We measured functional mobility, specifically gait, turning, and rising, with the Timed Up and Go (TUG). The TUG is scored as the number of seconds needed to rise from a chair, walk 10 feet, turn, and return to the seated position. The historical mean TUG score for PD populations is between 10.5–14.8 seconds [21,22]. Balance and gait were assessed with the Tinetti Mobility Test, which has strong psychometric properties and consists of 2 subscales: balance tests (9 items, range: 0–16) and gait tests (7 items, range: 0–12), where higher scores reflect better performance [23]. We also assessed balance and flexibility with the Functional Reach Test, which measures how far forward one can reach when bending from the waist [24]. The TUG, Tinetti Mobility Test, and Functional Reach Test are all recommended tests of these motor domains in PD [25]. Each study team member conducting assessments was trained according to test-specific instructions, with review of simulated participant assessments by the principal investigator.

Additional exploratory outcomes included mood and brief measures of cognition, as these are critical non-motor domains affected by PD which may be amenable to improvement with different exercise modalities. We used the Hospital Anxiety and Depression Scale (HADS), a brief, 14-item, highly validated scale, to measure anxiety and depression (7 items per subscore, each item scored 0–3), where scores >8 on either subscore indicate probable symptoms. To assess cognition, we administered the Digit Span Test (DST), testing the number of digits a participant can recall correctly after verbal presentation [26]. Both forward and backward digit span were measured, with raw scores scaled based on age. DST is a sensitive measure of working memory, a domain frequently affected in PD [27]. The Symbol Digit Modalities Test (SDMT) assesses visual scanning, divided attention, tracking, and motor speed, all of which can be affected in PD [28]. For the SDMT, the participant is presented with a reference key and has 90 seconds to pair specific numbers with given geometric figures, with either written or oral responses.

Following the ten weeks of karate classes, participants attended a post-intervention study visit at Rush University, where the following measures were repeated: Fall frequency, IPAQ, PDQ-8, TUG, Functional Reach Test, and Tinetti Mobility Test. Each objective mobility, balance, and gait assessment was administered by the same study team member, respectively, at the pre- and post-intervention visits to avoid inter-rater variability. The study team members (JF, BS, CN, ML, CW) administering the post-intervention assessments were blinded to pre-intervention assessment results and attendance records.

Six months post-intervention, a study team member telephoned all completers to determine whether they were still practicing karate, either in formal classes, on their own, or both. We assessed reasons for stopping, if applicable, and repeated the PGIC. A different team member than had interfaced with the participants at the pre- and post-intervention visits was selected for the follow-up calls to limit social desirability bias.

### Intervention

The intervention consisted of ten weeks of twice-weekly, non-contact, PD-specific karate classes in the Shotokan style, for a total of twenty classes, each one hour long. Participants attended group classes at one of two community karate studios in the Chicago suburbs based on location and schedule desirability. Each one-hour class followed a pattern of warm-ups, stationary basics, moving stance work, striking, conditioning, and as the curriculum progressed, kata (a traditional, choreographed series of movements), and mindfulness and breathing exercises. Details of the class curriculum are in S1 Appendix. Specifically, the warm-ups consisted of light stretching and joint mobility exercises. Conditioning included agility and coordination exercises, often striking a punching bag or practicing kicks with the help of the instructors. Kata is a traditional, choreographed sequence of movements that combines footwork, armwork, and movement across the floor. The kata elements were taught individually until the entire sequence was learned, and then the choreography was practiced at every class. At each class, students practiced core strengthening exercises on the floor and techniques for falling safely. Although breathing and mindfulness were incorporated subtly throughout all elements of class, each session concluded with a cooldown exercise that brought increased focus to these relaxation techniques. All classes were non-contact. Participants would practice strikes in the air, against a punching bag, or towards a pad held by an instructor.

Classes were led by karate instructors with at least one year of prior experience teaching karate to adults professionally, and who had themselves achieved a black belt in karate, demonstrating mastery. All instructors completed an interactive, in-person training with the principal investigator (JF) on common signs, symptoms, and complications of PD (S2 Appendix). Instructors were encouraged to ask questions, and all questions were answered prior to the beginning of the intervention. Training support continued as needed throughout the study. Two to three instructors and a study team member were present at each class and assisted participants as needed. Modifications were offered when necessary, e.g., if participants were experiencing imbalance, they were encouraged to broaden their stance and focus on arm movements, allowing them to continue participation in that day’s class. Participants were encouraged to attend all classes, and allowed to make up a missed class at the alternate studio location, however, there were no penalties for missed classes. The intervention is described in full as a TIDieR checklist, and attached as (S3 Appendix).

### Statistical analysis

This pilot study was intended to capture feasibility, and due to scheduling logistics of the karate studios, karate instructors, study team members, and participants, the class dates were pre-determined. Sample size was primarily defined by the number of individuals able to be recruited ahead of, and available to participate in, the 10-week summer session. Our power analysis was based on the TUG, assuming a mean score of 14.8 seconds and standard deviation of 5.8 seconds for historical PD populations, minimal clinically important difference of 5 seconds [21,22], correlation between baseline and follow-up assessments of 0.5, alpha of 0.05, and power of 0.9, yielding a sample size of 15.

Study data was collected and managed using REDCap, a secure, web-based, electronic data capture tool hosted at Rush [29]. Data was exported and analyzed in Stata 15 [30]. Anonymized data on pre- and post-intervention clinical characteristics, questionnaires, and assessments will be shared by request with any qualified investigator. We report the basic descriptive statistics of the cohort, and for feasibility, we report on dropout rate, adverse events, falls, and attendance, defined as the percentage of the 20 classes attended. We summarized categorical variables by frequencies and percentages, assessed continuous variables for normality, and summarized by mean and standard deviation, or median and interquartile range, as appropriate. We analyzed whether participants’ individual self-determined goals for the class were achieved, whether they had an interest in continuing karate classes, and whether they would recommend classes to others, with binary response options for each, respectively. We report on pre-intervention assessments, including IPAQ, TUG, Functional Reach Test, Tinetti Mobility Test, PDQ-8, HADS, DST, and SDMT.

We then analyzed the within-participant change in IPAQ, TUG, Functional Reach Test, Tinetti Mobility Test, and PDQ-8, HADS, DST, and SDMT, respectively, from pre- to post-intervention, using paired *t*-tests or nonparametric Wilcoxon signed rank tests, as appropriate. We calculated effect size (Cohen’s d) where appropriate, with <0.2 indicating trivial, 0.2–0.5 small, >0.5–0.8 medium, and >0.8 large effect sizes, respectively. As all participants chose only two of the five response options to the fall frequency question, (“Never,” “Rarely,” “Monthly,” “Weekly,” or “Daily), this was rendered a binary variable. We analyzed pre- and post-intervention fall frequency using McNemar’s test. Given the ordinal nature of the PGIC, we report on the percentage of completers endorsing each of the 7 response options at the post-intervention visit and six-month follow-up call, and compared the proportion indicating a clinically meaningful change (score of 5–7) at each time point using McNemar’s test to evaluate for a change in the proportion over time.

## Results

### Baseline characteristics of participants

**Fig 1:**
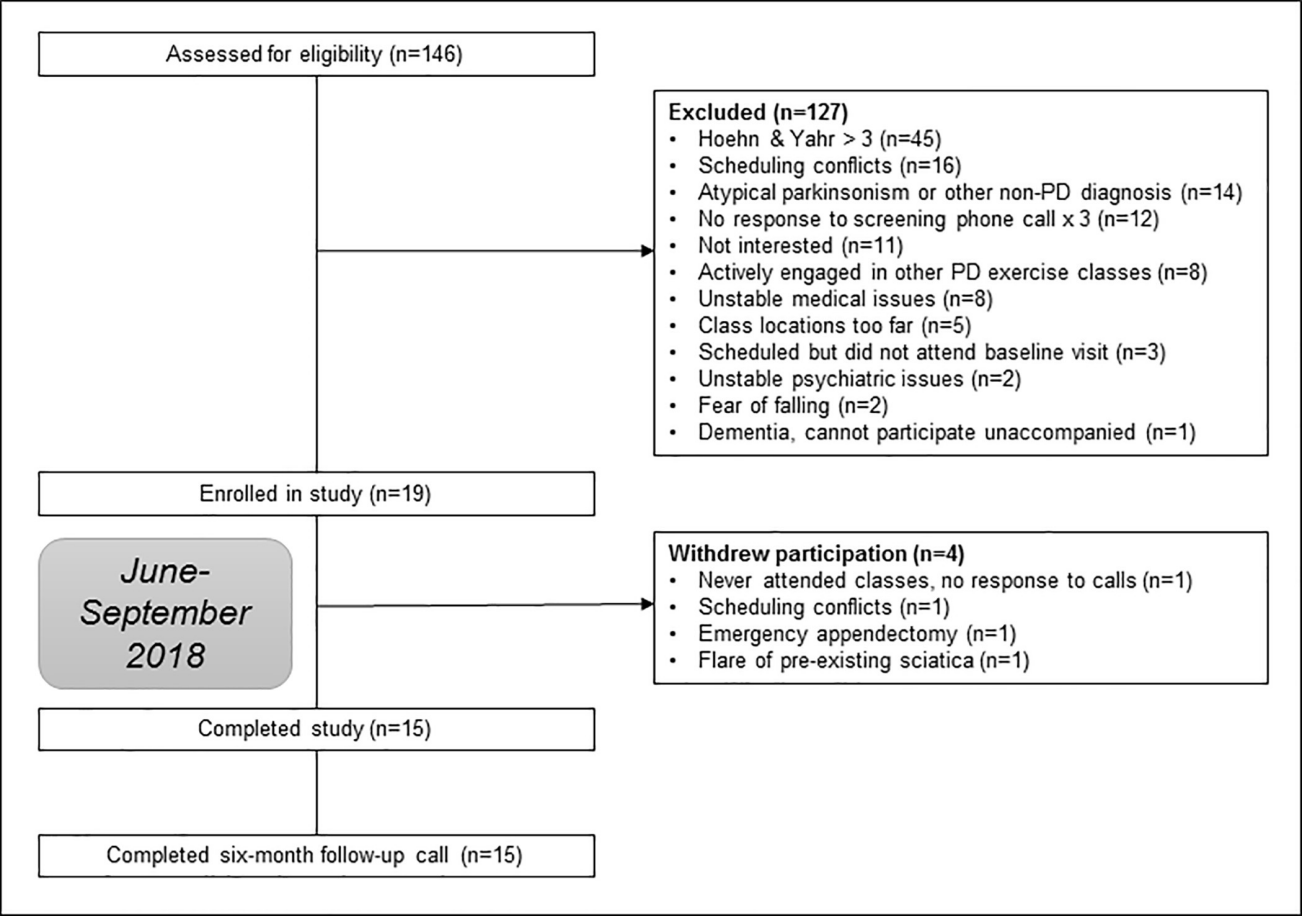
Study flow diagram. Study flow diagram demonstrating potential participants screened for eligibility, participants enrolled and completing the study, and reasons for ineligibility or exclusion.

We assessed 146 individuals for eligibility (Fig 1). Among 127 people excluded, the main reasons were: Hoehn and Yahr Stage >3 at the most recent outpatient visit or by self-report (n = 45), scheduling conflicts with karate classes (n = 16), atypical parkinsonism/non-PD diagnosis (n = 14), no response to screening phone calls (n = 12), or lack of interest (n = 11). Nineteen participants enrolled in the study and completed the pre-intervention visit, 15 from Rush University’s Parkinson’s Disease and Movement Disorders Program, and 4 recruited from outside neurologists. Table 1 demonstrates the baseline characteristics of all participants (n = 19) and all participants completing the study (n = 15). Based on the IPAQ, participants reported a mean of 5.37 days in which they walked at least 10 minutes at a time, with a median of 30 minutes spent walking on each such day (interquartile range 30–90 minutes), and a mean of 287 minutes per day spent sitting (SD 203) in the preceding week. Participants reported engaging in vigorous physical activity, such as aerobics, fast cycling, or heavy lifting, on 2.17 days per week (SD 2.18) and for a mean of 51.25 minutes (SD 31.59) on each such day. Participants also reported engaging in moderate physical activity, such as carrying light loads or cycling at a regular pace, on 2.29 days per week (SD 2.43) and for a mean of 88.89 minutes (SD 81.42) on such days.

**Table 1:** Baseline characteristics of KICK OUT PD cohort.

	Participants Enrolled n = 19	Participants Completing Study n = 15
**Female, %**	**52.6**	**53.3**
**Mean age, years (SD)**	**63.1 (10.8)**	**63.9 (11.1)**
**Race, %**		
**Caucasian** **Asian** **Declined to answer**	**84.2** **5.3** **10.5**	**80** **6.7** **13.3**
**Hispanic, %**	**10.5**	**13.3**
**Education, %** **Some college or trade school** **Bachelor’s degree** **Master’s degree** **Professional or doctorate degree**	**10.5** **26.3** **36.8** **26.3**	**13.3** **26.7** **40** **20**
**Median PD duration, years (range)**	**6 (2–20)**	**6 (2–20)**
**Hoehn & Yahr Stage, %** **2 –bilateral disease without postural instability** **3 –bilateral disease with postural instability**	**94.7** **5.3**	**93.3** **6.7**
**Status post deep brain stimulation, %** **Unilateral** **Bilateral**	**5.3** **5.3**	**6.7** **6.7**
**Fall frequency in the past 12 months, %** **Never** **Rarely**	**52.6** **47.4**	**46.7** **53.3**
**Prior karate participation, %**	**15.8**	**13.3**
**Physical activity level in the prior 7 days (IPAQ)** **Mean days, vigorous physical activity** **Mean days, moderate physical activity** **Mean days, walking at least 10 minutes at a time** **Median time walking each day, minutes (IQR)** **Mean time, sitting on a daily basis, minutes**	**2.2 (2.2)** **2.2 (2.4)** **5.4 (1.9)** **30 (30–90)** **286.7 (203.3)**	**2.5 (2.3)** **2.29 (2.4)** **5.33 (2.0)** **35 (30–90)** **264 (194.8)**

SD: Standard deviation. PD: Parkinson’s Disease. IQR: Interquartile range. IPAQ: International Physical Activity Questionnaire. IQR: Interquartile range.

### Feasibility outcomes

Fifteen participants completed the post-intervention assessments. Among those lost to follow-up, two were due to non-intervention-related illness or injury (appendicitis and pre-existing sciatica, respectively), one due to unexpected scheduling conflicts, and one withdrew prior to starting classes without providing a reason, yielding an overall 21.1% attrition rate. Neither the participant with appendicitis nor the participant withdrawing due to pre-existing sciatica felt that these were adverse effects due to karate, nor did the study team find them to be related. There was no change in fall frequency during karate classes, with the percentage of participants reporting rare falls prior to the study(vs. no falls) decreasing from 53.3% pre- vs. 20% during the study (McNemar’s test statistic = 5, exact p = 0.06). As shown in Table 1, there were no significant differences in sample characteristics between those who withdrew compared with those who completed the study (p = 0.86–1.00). Mean attendance at twice-weekly classes was 86.7% (SD 9.9%).

After 10 weeks, 100% of the 15 remaining participants intended to continue karate, 100% would recommend the classes to a friend with PD, and 100% felt they had achieved their pre-intervention personal goal, respectively. These personal goals revolved around themes including improvement of balance, mobility, and overall improved management of PD symptoms; benefiting from the social aspect of committing to exercise at a time and place with other people; improved mindfulness, and more general goals of having fun, getting exercise, and working towards a better future for themselves. Detailed qualitative analyses of pre- and post-intervention expectations, achievements, facilitators, and barriers are forthcoming.

### Change in pre- and post-intervention outcomes

We found a statistically and clinically significant improvement in quality of life of 5.9 points on the PDQ-8 (25.3 vs. 19.3, mean difference -5.9, 95% CI -9.9 to -2, p = 0.01, effect size 0.8, Table 2). There were no changes in physical activity levels compared to baseline, as measured by the IPAQ and shown with additional exploratory outcomes in Table 2. Mean TUG decreased from 9.6 to 9.0 seconds (mean difference -0.6, 95% CI -1.3 to 0.2, p = 0.12, effect size 0.4), however this population was notably faster at baseline compared to historical mild- to moderate-stage PD validation cohorts. We found no change in Functional Reach Test, and a small change in Tinetti Mobility Test.Additional exploratory outcomes are shown in Table 2: Mean TUG improved from 9.6 to 9.0 seconds (mean difference -0.59, 95% CI -1.34 to 0.17, p = 0.12, effect size 0.43), however this population was notably faster at baseline compared to historical mild- to moderate-stage PD validation cohorts. We found no change in Functional Reach Test (10.8 vs. 12.5 inches, mean difference 1.73, 95% CI -2.5 to 5.96, p = 0.39, effect size 0.23), and a statistically significant change in Tinetti Mobility Test (27.07 vs. 27.93, mean difference 0.87, 95% CI 0.24 to 1.49, p = 0.01, effect size 0.77).

**Table 2:** Pre- and post-karate secondary outcomes among participants.

n = 15	Pre-intervention	Post-intervention	Mean difference (95% CI)	Effect size	t-statistic	p-value
**Patient-Reported Outcomes**						
Parkinson’s Disease Questionnaire-8	25.3 (20.8)	19.3 (19.6)	-5.9 (-9.9, -2)	0.83	-3.23	**0.01**
**Balance & Mobility Assessments**						
Timed Up and Go, seconds*	9.6 (2.2)	9.01 (1.9)	-0.6 (-1.3, 0.2	0.43	-1.66	0.12
Functional Reach Test, inches	10.8 (2.4)	12.5 (7.0)	1.73 (-2.5, 6.0)	0.23	0.88	0.39
Tinetti Balance and Mobility Test	27.1 (1.0)	27.9 (0.3)	0.9 (0.2, 1.5)	0.77	2.98	**0.01**
**Patient-Reported Physical Activity (International Physical Activity Questionnaire)**						
Days of vigorous activity^1^	2.5 (2.3)	2.4 (2.0)	-0.1 (-1.6, 1.5)	0.03	-0.1	0.92
Minutes of vigorous activity per day^7^	51.3 (31.6)	69.4 (45.3)	18.1 (-3.7, 40.0)	0.70	1.97	0.09
Days of moderate activity^1^	2.3 (2.4)	2.7 (2.6)	0.4 (-0.9, 1.7)	0.19	0.7	0.49
Minutes of moderate activity per day^6^	88.9 (81.4)	82.2 (91.5)	-6.7 (-64.4, 51.0)	0.09	-0.27	0.8
Days with >10 minutes walking	5.3 (2.0)	5.8 (1.4)	0.5 (-0.7, 1.6)	0.23	0.89	0.39
Minutes of walking per day	60.3 (48.5)	63.0 (77.8)	2.7 (-49.6, 54.9)	0.03	0.11	0.91
Minutes sitting per day^2^	304.6 (175.7)	238 (190.3)	-66.6 (-183.4, 50.1)	0.34	-1.24	0.24

All data reported as mean (standard deviation), unless otherwise indicated. Bolded values indicate statistical significance, two-tailed p < 0.05. Superscripts indicate the number of missing values, and mean (SD) is reported only for patients with both pre-and post-intervention scores. Effect sizes categorized as: <0.2: trivial; 0.2 - <0.5: small; 0.5–0.8: medium; >0.8: large. Parkinson’s Disease Questionnaire-8: Scored 0–100; higher scores signify worse quality of life. Timed Up and Go: Seconds to rise from chair, walk 10 feet, turn, return to seat; historical PD mean: 10.5–14.8. Functional Reach Test: How far forwards an individual can reach, in inches, when bending from waist. Tinetti Balance and Mobility Test: Total range 0–28, where higher = better function.

There were no significant changes in the exploratory outcomes of mood or cognition. The mean HADS depression subscore was 4.47 (SD 4.32) pre-intervention, and 4.8 (4.13) post-intervention (p = 0.53). The mean HADS anxiety subscore was 5.53 (3.81) pre-intervention and 5.93 (4.67) post-intervention (p = 0.35). Neither the scaled digit span forward score (12.33, SD 3.37 pre-intervention vs. 11.93, SD 3.65 post-intervention, p = 0.41) nor the scaled backward score (10.67, SD 2.97 pre-intervention vs. 10.47, SD 2.53 post-intervention, p = 0.81) demonstrated change. Similarly, there was no change in the SDMT scaled score (pre-intervention: mean 96.17, SD 9.73; post-intervention 95.67, SD 10.87; p = 0.78).

### Global impression of change and sustainability

**Fig 2:**
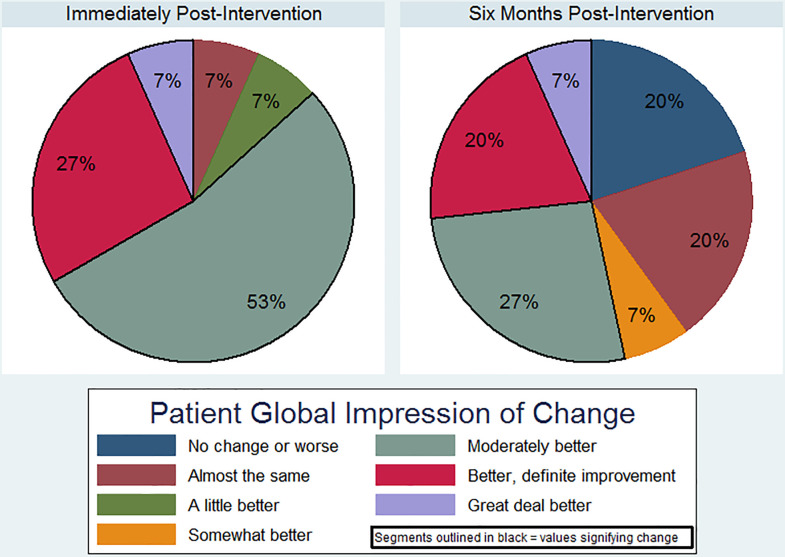
Global impression of change. Participants’ global impression of change immediately post-participation and six months post-participation.

Fig 2 shows the percentage of participants endorsing each response option on the Patient Global Impression of Change, with outlined segments indicating clinically meaningful change. Overall, 86.7% of completers endorsed clinically meaningful change immediately post-intervention, and 53.3% did so six months later (McNemar’s test p = 0.03). After six months, 8/15 individuals (53.3%) continued practicing karate, with seven still attending classes (mean 1.58 days per week, SD 0.79). Five individuals continued to practice karate on their own for a mean of 3.6 days per week (SD 2.07), 22 minutes per session (SD 8.37). Surprisingly, five participants (33%) competed in a regional tournament during follow-up and all advanced to the next level of training (i.e., from white to yellow belt). Among those who did not continue karate, three cited a lack of time, two cited inconvenient locations, and one each reported classes to be too costly or of insufficient benefit, respectively.

## Discussion

Individuals with PD demonstrated strong adherence to a twice-weekly, 10-week karate exercise program, no adverse events or change in fall frequency during classes, and 53% sustained participation six months after the study intervention ended. Participants achieved their individual pre-intervention goals and experienced a significant improvement in quality of life and overall wellbeing. However, we found no change in activity level, objective mobility, or exploratory mood and cognitive outcomes. We found no change in self-reported physical activity levels between pre- and post-intervention, indicating that karate may have substituted for prior activity rather than adding to baseline levels. Six months after the intervention, over half of participants continued to practice karate and to endorse meaningful changes in wellbeing compared to baseline. Of those who stopped karate after study completion, the majority cited scheduling logistics or financial cost as the barriers to participation. Only one participant chose to discontinue because of perceived lack of benefit. Our results support the preliminary feasibility, safety, and sustainability of karate classes tailored to individuals with PD, however despite achievement of individually-set goals and improvement in quality of life, objective benefits were not demonstrated.

In a recent German pilot study comparing weekly karate vs. dance among individuals with PD, participants self-selected into karate training, dance training, or a sedentary control group, with 16, 9, and 12 participants in each, respectively [12]. Dahmen-Zimmer et al. highlighted the feasibility of conducting karate classes with PD patients and reported high adherence as a marker of high acceptability. Here, we have replicated those findings in a US cohort. In the German study, small improvements were detected in emotional, cognitive, and motor performance compared with the sedentary controls, however there were no differences between the karate and dance groups. In that study, attrition (36%) was greater and mean attendance (83%) was less than in our study, 21% and 87%, respectively. While the prior study lasted 30 weeks—which may account for the higher attrition and lower adherence—there was no follow-up after the intervention ended. Notably, the groups in the German study differed by gender, with the authors hypothesizing that karate seemed more appealing to men than women. In our study, 52% of participants were female, suggesting karate may be more gender neutral and enhancing the potential for scalability. Interestingly, the German study allowed care partners to participate in the exercise modalities but did not analyze or control for this potential confounder. While valuable as the first study of karate, the conclusions in the German paper focused on demographic differences between the self-selecting groups. In this first-in-the-US study, we focus on feasibility of this intervention in order to plan for a larger, controlled trial.

Although exploratory, this is the first report to our knowledge to demonstrate an improvement in PD-specific quality of life from karate, with the pre-post change in the PDQ-8 meeting the minimum clinically important difference of a 5.9-point decrease [31]. While there was no significant change in physical activity level nor objective mobility assessments, we hypothesize that the improved quality of life may stem from two potential sources, which require further investigation. First, our power analysis was based on the TUG, however this population was notably faster at baseline compared to the historical mild- to moderate-stage PD validation cohorts [21,32]. The minimum clinically important difference in the TUG ranges from 2–5 seconds in PD populations. Thus, we were underpowered to detect a change in mobility with this assessment, and participants may have achieved motor benefits that were too subtle to detect using the TUG, particularly when starting from a near-normal baseline. This hypothesis is supported by the participants’ pre-intervention individual goals being largely focused on mobility and balance, with a 100% rate of self-reported goal achievement post-intervention.

Additionally, post-intervention qualitative feedback from participants highlighted the value of learning and practicing how to fall during class. While fear of falling was not specifically assessed, and participants in this cohort rarely fell at baseline, falls significantly impact quality of life in individuals with PD [33]. Reciprocally, worse quality of life and a history of prior falls predict fall frequency [34,35]. In prior PD studies, tai chi interventions have improved balance and mobility, and reduced fall risk [7]. Although distinct from tai chi due to more aerobic activity and faster movements, karate similarly integrates flexibility, balance, focused attention, and patterned movements, suggesting a similar potential to reduce fall risk. Because we assessed baseline fall frequency over the preceding 12 months, and again at the post-intervention visit for the preceding 10 weeks of karate classes only, comparisons of fall frequency rate are biased by the different time frames. Our goal with asking in this way was to detect safety concerns directly related to the karate classes. Karate training in older non-PD cohorts has improved motor reactivity, stress tolerance, and gait, even under dual-task conditions [36,37]. In the current study, karate instructors enhanced the class curriculum with PD-focused training on how to fall safely and how to return to standing. We hypothesize that karate may have contributed to subjective improvements in balance and mobility that we were underpowered to detect, and/or mitigated fear of falling, which we did not set out to measure a priori. These hypotheses require further testing.

Social connections formed in group-based exercise may also have significant implications for quality of life in people with PD. We suspect that this additional unmeasured factor may contribute to both the gains in quality of life and wellbeing, along with the high adherence during the study and the rate of continued post-intervention participation [38]. We are actively investigating the impact of camaraderie during group exercise for PD in ongoing work.

We did not detect changes in the exploratory outcomes of mood and cognitive domains. The ten-week duration of the trial itself likely rendered measurable change in these domains highly unlikely, compounded by the study being underpowered. Improvements have been seen in such domains in prior karate studies, albeit of longer duration [10,12].

## Limitations

This pilot was an open-label, single-center study without a control group, and thus our results should be interpreted with caution given the potential for a placebo response to a novel, brief intervention. Many potential participants were excluded from our recruitment population, with disease severity as the most common reason (Hoehn and Yahr Stage >3) followed by scheduling conflicts and lack of interest. To the former, we limited pilot participation to individuals with milder disease given safety concerns and unknown fall risks. However, the study team is actively working with karate professionals to design and test adapted curricula for individuals with more advanced disease. While many participants were excluded due to scheduling conflicts or distance from the two karate studios involved, limiting external validity, this reflects the pilot nature of the study. We were necessarily restricted to a limited number of heavily supervised sites. Logistic barriers to participation may be attributable to the employment status and large catchment area from which our clinic population arises. The number of participants excluded based on lack of interest may indicate the barriers faced by many with incorporating new exercise routines, or simply that karate may only interest a select few, Yet as of 2018, there were over 75,000 martial arts studios throughout the US, though we are unaware of many offering PD-specific classes beyond our pilot study [39]. We suspect that—similar to the growing implementation of boxing programs for individuals with PD across the globe—the provision of PD-specific instructor training and adapted class curricula could promote dissemination into communities, overcoming the logistical barriers encountered herein [40].

We anticipated enrolling more individuals with moderate PD (Hoehn and Yahr Stage 3, moderate bilateral symptoms with postural instability), and powered our study based on published PD-specific TUG performance including that population. However, because of slower than anticipated recruitment and time constraints related to studio and study staff availability, we opted to launch the study with a sample size of 19 rather than delaying launch and shortening the study duration in an attempt to reach our target sample size of 30. Furthermore, our participants had predominantly mild PD (Hoehn & Yahr stage 2) and performed far better at baseline than anticipated (mean of 9.6s on TUG compared to 14.8s for historical controls). Despite improvement by a mean of 0.6s—approaching non-PD comparators—this unexpectedly high baseline level of performance, and attrition of 4 participants, yielded a study powered at only 19.9% to detect a clinically meaningful change in TUG. Although pre- and post-intervention TUG scores were highly correlated (correlation coefficient = 0.79, p = 0.0004), as were the pre- and post-intervention PDQ-8 scores (correlation coefficient = 0.94, p < 0.0001), this does not overcome the limited power. Also, 15.8% of the participants had previously participated in karate classes, which may have conferred some degree of familiarity compared with those who had not practiced before. However, among those endorsing prior participation, all had participated briefly and only during early childhood, thus we suspect that skill retention or carryover was minimal. An additional limitation is that we did not measure exertion during class and future studies could be strengthened by monitoring heart rate and/or asking participants to rate perceived exertion during classes in order to assess the challenge and vigor of this particular exercise. Finally, to minimize participant burden, our assessments did not include PD-specific mobility examinations, such as the Movement Disorders Society-Unified Parkinson’s Disease Rating Scale, nor assessment of dyskinesias or motor fluctuations. Given the overrepresentation of mild PD in the study, dyskinesias and fluctuations would be expected to contribute minimally, however a larger, longer, more representative, adequately powered, randomized controlled trial is needed to better delineate the effects of karate in PD. While the TUG, Tinetti, and Functional Reach tests are recommended by the Movement Disorders Society as instruments to assess posture, gait, and balance in PD, more comprehensive future evaluations should include the UPDRS-derived postural instability and gait difficulty score, instrumented examinations of balance and gait, and patient-reported outcomes, such as the Falls Efficacy Scale and Fear of Falling in the Elderly-Modified.

## Conclusion

In summary, we demonstrated the feasibility of a brief, twice-weekly, community-based karate intervention for a cohort of individuals with primarily mild PD. Adherence to the intervention was high without adverse effects or increased falls, and participants experienced significant improvements in quality of life and overall wellbeing, along with achieving their pre-intervention individual goals for karate. However, we found no changes in mobility, physical activity, mood, or cognition. Despite the lack of objective benefit, over 53% of completers remained engaged in karate six months later, and one third advanced to the next level of training, highlighting potential sustainability at the individual level. These results support our efforts to investigate karate in a larger, randomized controlled trial, adequately powered to study mobility with validated, PD-specific assessments. Across populations of older adults, community-based group exercise programs have yielded health benefits and sustained adherence when they have been tailored to older adults’ needs: accessibility, affordability, socialization, and when led by caring instructors [41]. As with medication regimens, it is unlikely that a single form of exercise will be acceptable, beneficial, and sustainable for all individuals with PD. However, if karate proves to be an engaging, safe, adaptable, and positive option, there is strong potential for dissemination given the distribution of martial arts studios, expanding the menu of options for individuals with PD to become more active.

## Supporting information

S1 Appendix KICK OUT PD class curriculum. (PDF)

S2 Appendix KICK OUT PD instructor training. (PDF)

S3 Appendix KICK OUT PD intervention description TIDieR checklist. (DOCX)
