# Supplementary material for: KICK OUT PD: Feasibility and quality of life in the pilot karate intervention to change kinematic outcomes in Parkinson’s Disease
Source: PLoS One. 2020 Sep 9;15(9):e0237777. doi: 10.1371/journal.pone.0237777 (PMC7480843; doi:10.1371/journal.pone.0237777)
Supplement: S1 Appendix — (PDF) [file pone.0237777.s001.pdf]

Kick Out PD - Curriculum Overview

Warm-ups; Basics; Blocks & Strikes; Mitt Work/Ladder Drills; Break Falls; Meditation/Cool-down):

Warm-up (10 minutes):
Gripping ground with feet
Opening and closing fists
¼ or ½ knee bends
Hip circles
Flexion and extension with the spine
Body rotation
Arm side swings
Arm circles
Rotator cuff
Stretching Neck sideways with one hand
Side Bends reaching arm extended to one side
Single knee ups
Double knee ups
Marching in place
Squats with arms extended
Lunges (big)
Lunges with twist (Charlie's Angels)
Shuffle sideways (wide) – two steps left and two steps right

Kihon (Basics) from Heiko Dachi (feet shoulder width apart, knees slightly bent) -- (10 minutes):
Deep breathing with palms extended slowly out and back. Then fast.
Double Teisho uchi (palm strike)
Single Teisho uchi (palm strike alternating arms)
Tsuki (Single punch alternating arms)
Age Uke (Rising block on two count)
Age Uke (Rising block on one count)
Gedan Barai (Downward block same arm with other hand on hip)
Gedan Barai (Downward block - alternating arms)
Hiza Geri (Knee strike alternating legs)

Dachi – Stance Work (10 minutes):
Zenkutsu Dachi – Mae (Front stance - moving forward)
Zenkutsu Dachi – Sagatte (Front stance - moving backward)
Teisho Uchi – (Double palm strike slowly on two count, then on one count)
Oizuki – (Front punch on two count, then on one count)
Age Uke – (Rising block on two count, then on one count)
Gedan Barai – (Downward block on two count, then one count)
Hiza Geri – (Knee strike - forward only)

Tsuki/Uchi – (Strikes from Fighting Stance - switch feet and repeat) (10 minutes):

Kizame Zuki (Jab - stationary)
Gyaku Zuki (Reverse punch - stationary)
Kizame-Gyaku Zuki (Jab-Reverse punch - stationary)
Kizame Zuki (Jab) with front foot step
Gyaku Zuki (Reverse punch) with front foot step
Kizame-Gyaku Zuki (Jab-Reverse punch) with single front foot step

Mitt/Paddle Work and Footwork on Ladders or over Hurdles (10 minutes):

Do a footwork exercise thru the ladder or over the hurdles and run down to strike paddles (2 rounds each side of each footwork drill – high knees, step in and out, high knees sideways, step in and out sideways)

Target Drills:

1. Kizame Zuki (Jab on mitt stationary and flashing for reaction)
2. Gyaku Zuki (Reverse punch on mitt stationary and flashing for reaction)
3. Kizame-Gyaku Zuki (Jab-Reverse on mitt stationary and flashing for reaction)
4. Age Uke -Gyaku Zuki – (Rising Block - Reverse Punch on mitt flashing for reaction)
5. Gedan Barai – Gyaku Zuki (Downward Block – Reverse Punch on mitt flashing for reaction)

Break Falls (5 minutes):

Lay down and tuck knees to chest while rocking; practice side and back falls while on your back --- students can rock up as high as they feel comfortable. As you roll back slap both hands on the mat palms down next to your thighs while keeping your chin connected to your chest. Always prevent your head from touching the ground. Then practice standing up correctly. Roll to your side and then to all fours on your hands and knees. One knee up at a time, with both hands on knee that is up. Then stand up.

Cool-down / Meditation (5 minutes):

Static Stretches (One leg in, one leg out; both legs out; legs in V; butterflies; cat stretch; etc)

Meditation --- Students should focus on breathing (deep, slow-controlled breaths). Discuss the importance of deep breathing to de-stress, calm the mind and to focus on the task at hand. Breath work should be done on a count of 4 for the inhale and a count of 4 for the exhale. Place your hands on your stomach and practice for deep breathing for 1 minute, while inflating the stomach on each inhale and deflating the stomach on each exhale.

Weekly Syllabus

Week 1–Basics (Rising Block, Downward Block, Front Punch, Knee Strike); Jab/Reverse Punch from fighting stance

Week 2 – Introduce Front Kick (use wall or it can be done from the floor on your back); Double Punch from Shoulder Width Stance; Jab and Reverse Punch from Front Stance; Break Falls from seated position.

Week 3 – Introduce Inward Block; Inward Block Reverse Punch (sword and shield); Break falls from squat position

Week 4 – Introduce Outward Block, Knee Strikes on targets, and 1st Four moves of Taikyoku Shodan;

Week 5 – Introduce Front Kick – Front Punch Combo and 1st 8 moves of first Kata

Week 6 – Introduce Roundhouse Kick and 1st 12 moves of first Kata

Week 7 – Introduce Side Kick, Ducking against punches, and 1st 16 moves of first Kata

Week 8 – Introduce Hammer Fist Strike, Target sparring and All of Taikyoku Shodan on instructor's count

Week 9 – Review of Taikyoku Shodan on count with emphasis on ¼, ½, and ¾ turns

Week 10 – Free Kata. Students perform the kata in its entirety, on their own with no counting.
