# Supplementary material for: KICK OUT PD: Feasibility and quality of life in the pilot karate intervention to change kinematic outcomes in Parkinson’s Disease
Source: PLoS One. 2020 Sep 9;15(9):e0237777. doi: 10.1371/journal.pone.0237777 (PMC7480843; doi:10.1371/journal.pone.0237777)
Supplement: S2 Appendix — (PDF) [file pone.0237777.s002.pdf]

KICK-OUT PD:

An Introduction to Parkinson's Disease and a New Intervention to Fight It

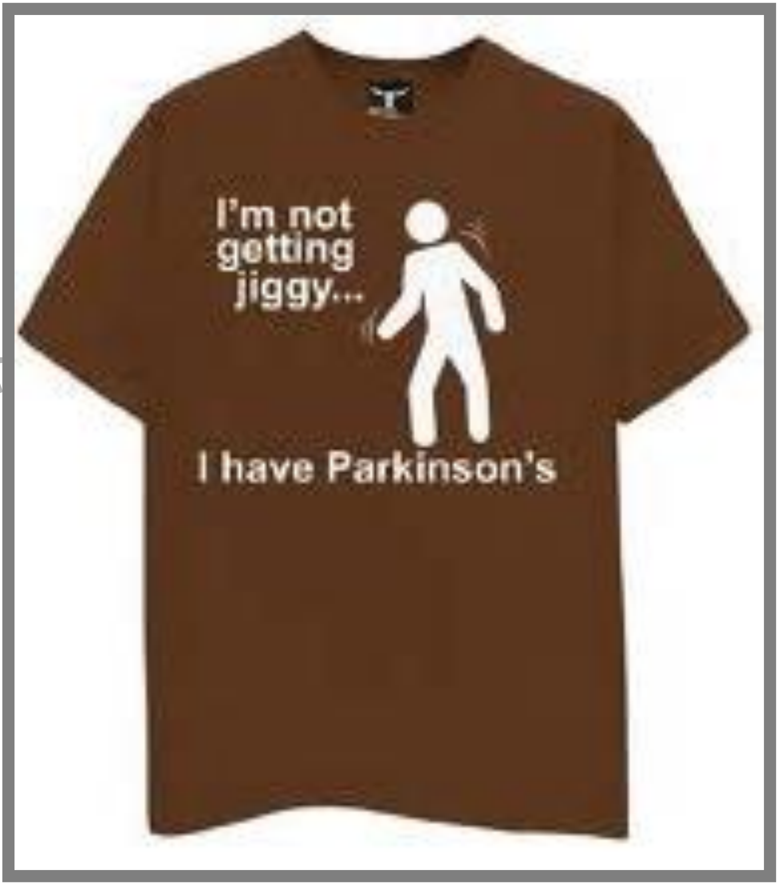

Jori Fleisher, MD MSCE
Assistant Professor of Neurological Sciences
Rush University Medical Center

Copyright pending – do not repr

Overview

- What is Parkinson's Disease?
  - Who gets it and when
  - Motor symptoms
  - Non-motor symptoms
- Life with PD
- KICK-OUT PD Study

Copyright pending – do not reprodu

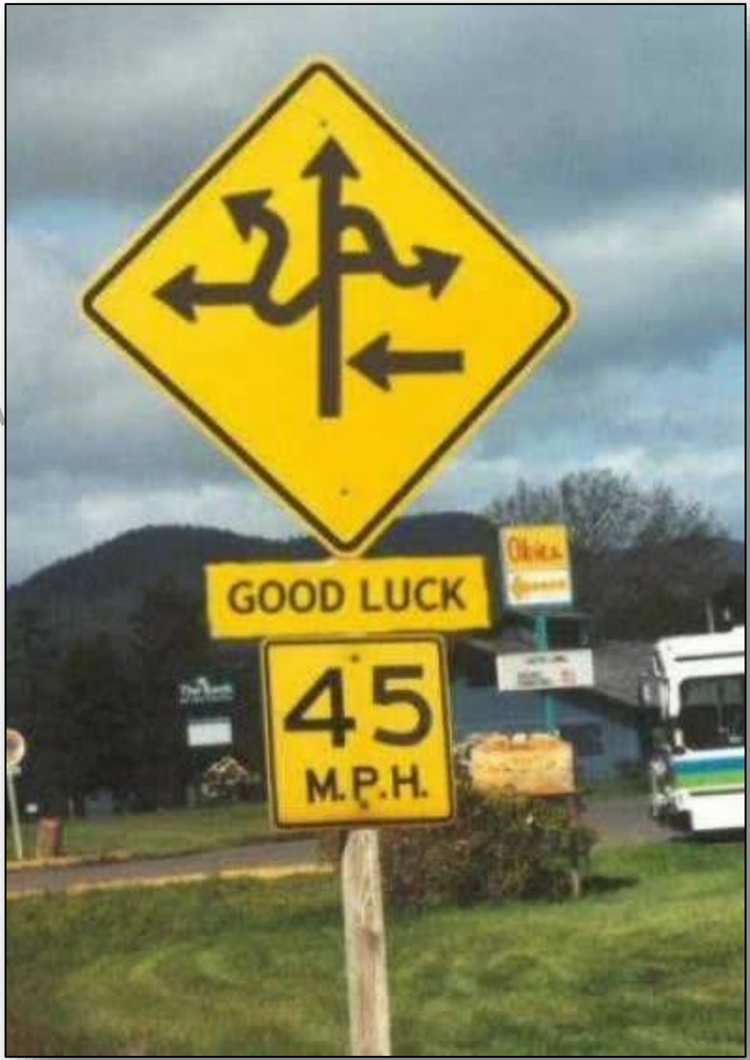

Parkinson's Disease

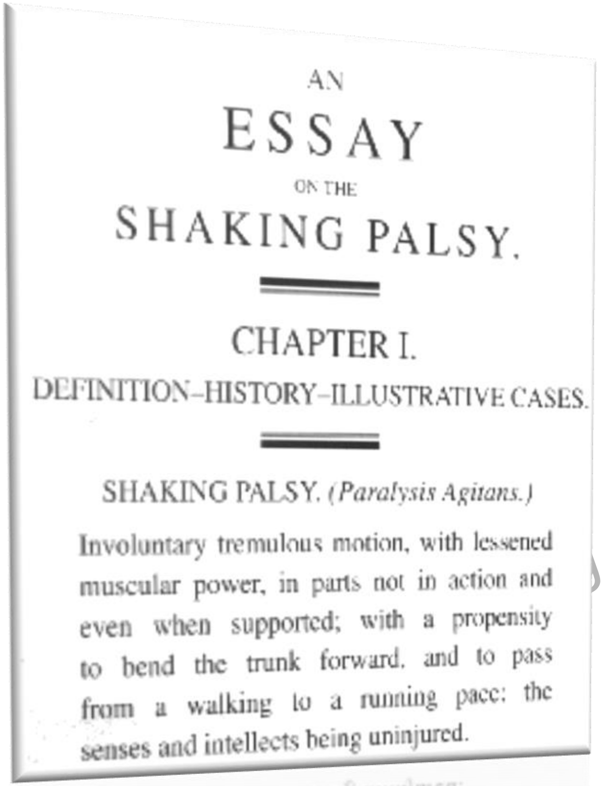

AN
ESSAY
ON THE
SHAKING PALSY.

CHAPTER I.
DEFINITION–HISTORY–ILLUSTRATIVE CASES.

SHAKING PALSY. (*Paralysis Agitans.*)

Involuntary tremulous motion, with lessened muscular power, in parts not in action and even when supported; with a propensity to bend the trunk forward, and to pass from a walking to a running pace: the senses and intellects being uninjured.

- **First described in 1817, based on SIX patients!**
- 2nd most common neurodegenerative disease
  - 1-2% of ppl >65y, 3-5% over age 85
  - 1-2 million people in the US & in Europe
  - More common in men (M:F ~1.5)
- **Progressive** over time
  - Symptoms and time course are HIGHLY VARIABLE
  - Every person with PD is different, though the vast majority will SLOWLY WORSEN over time
- **Incurable** at this time

g – do not reproduce without permission

Parkinson's Disease

- Symptom onset most often in 50s-60s
- Brain cells that produce dopamine start to die off prematurely
  - Dopamine involved with initiating and controlling movement; also with reward pathways and motivation
- Numerous medications and surgical options
  - Many symptoms can be managed much better and for much longer than ever before
- For most people, does not affect life expectancy

Copyright pending – do not reproduce without permission

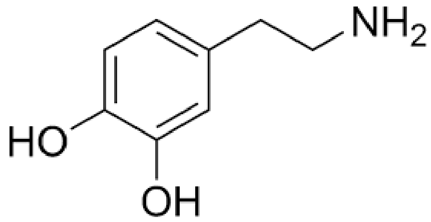

PD Clinical Features

- **Diagnostic features of *parkinsonism*:**
  - Slow, small movements (Akinesia/bradykinesia)
  - PLUS ONE of the following
    - Tremor (resting, 4-6Hz)
    - Rigidity
    - Postural instability

Tremor

Rigidity

Akinesia

Postural instability

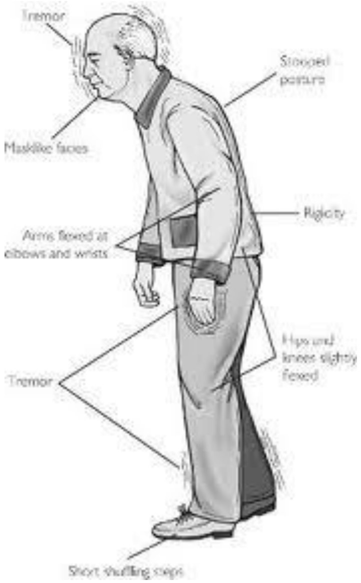

Copyright pending – do not reproduce without permission

PD Clinical Features

- Supportive criteria for the diagnosis
  - Meets criteria for parkinsonism PLUS
    - Begins on one side
    - Stays asymmetric
    - Progressive disorder
    - Response to levodopa
    - Gait problems: freezing, shuffling, falling
    - Stooped posture
    - Quiet, rushed voice
    - Decreased facial expression
    - Small, illegible handwriting (micrographia)

Copyright pending – do not reproduce without permission

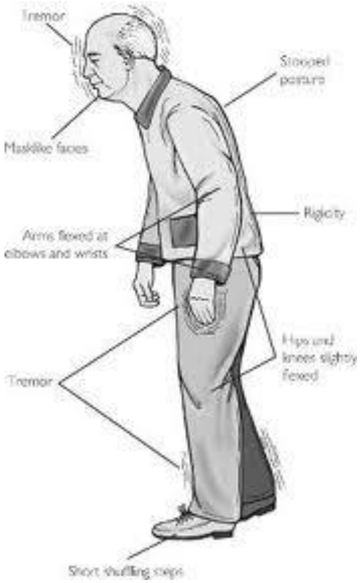

Motor & nonmotor manifestations

- Motor:
  - T.R.A.P.
- Nonmotor:
  - Sleep disturbances
  - Fatigue
  - Depression & Anxiety
  - Apathy
  - Cognitive changes and dementia
  - Pain & sensory changes
  - Quieter, hoarser, more strained voice
  - Constipation
  - Autonomic dysfunction (low blood pressure, dizziness or passing out)
  - Drooling, trouble swallowing, choking

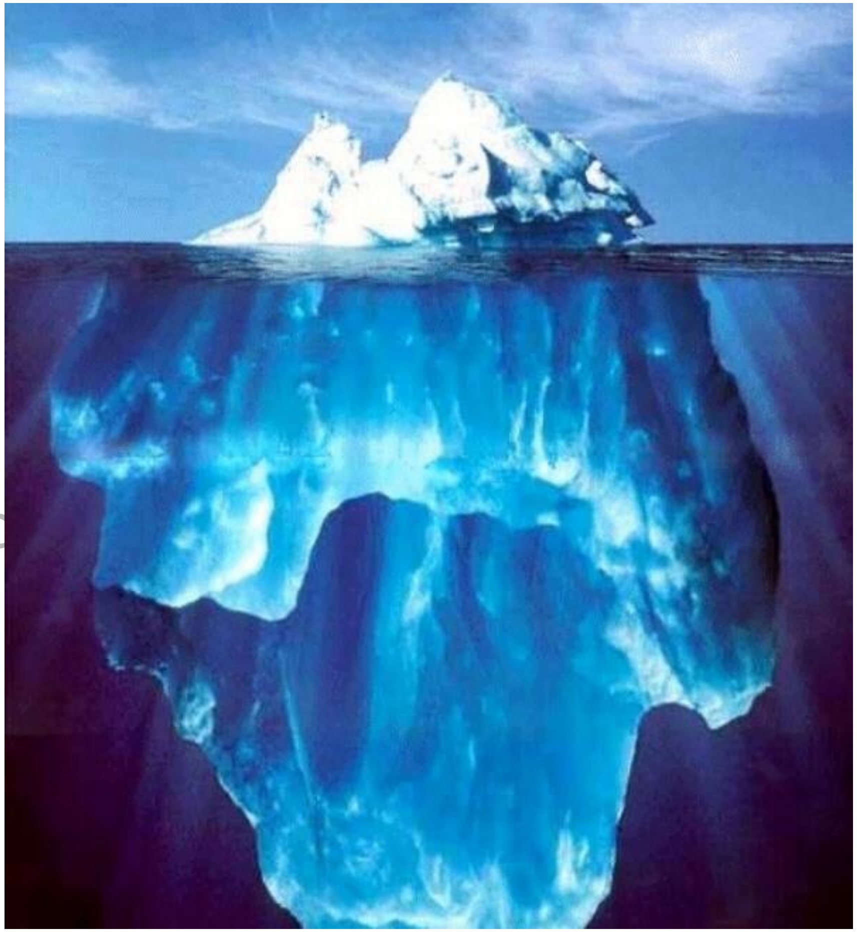

Copyright pending – do not repro

Medication vs. Exercise

- Medication:
  - Covers up the symptoms
  - Disease progresses unchanged

- Exercise:
  - Covers up the symptoms
  - *May change how quickly disease progresses*

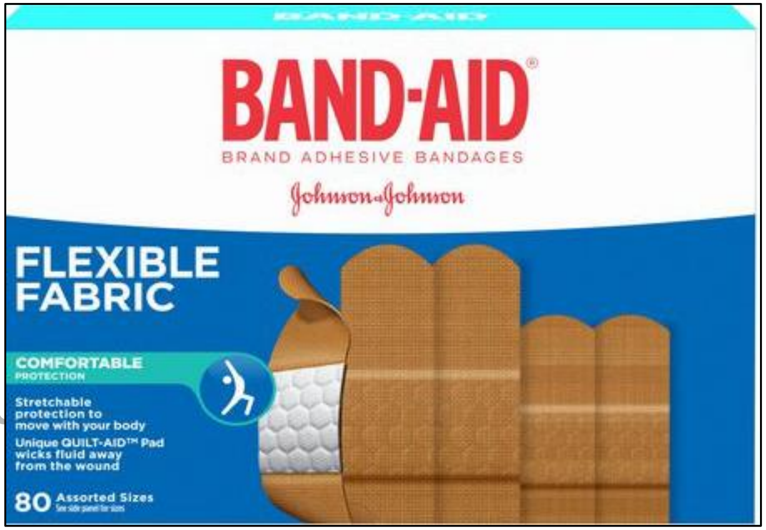

Copyright pending – do not reprodu

Cognitive changes

- >30% of people with PD have some degree of cognitive change in specific areas
- Things that can be affected:
  - "Tip-of-the-tongue" phenomenon
  - Attention, multitasking
  - Active memory
  - Executive function
  - Visuospatial function
  - Speed of thinking

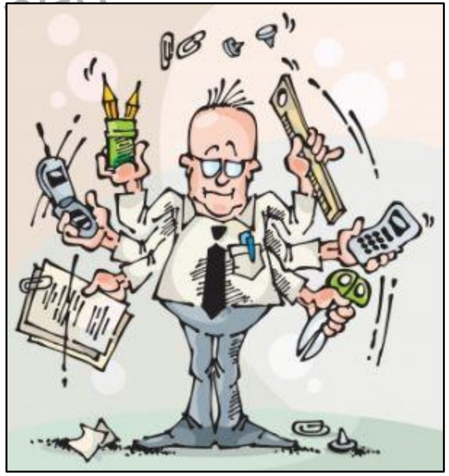

Copyright pending – do not reproduce without permission

Autonomic changes

- Orthostatic hypotension:
  - After sitting or lying for prolonged period, blood pools in legs.
  - Normal reflex on sitting/standing: veins constrict, push blood back to center of body
  - PD: reflex is blunted; lightheaded or dizzy on standing too quickly, brain not receiving enough blood flow
- How to help:
  - SLOW transitions between lying/seated and standing positions
  - If symptomatic, return to horizontal position, slowly transition again
  - Adequate hydration is key!
- Excessive sweating:
  - Less common but possible; more of a nuisance and dehydration risk; encourage adequate hydration!

Copyright pending – do not reproduce without permission

Parkinson's Disease

- Late symptoms
  - Motor fluctuations (on/off phenomena)
  - Freezing
  - Loss of drug effect, drug failures
  - Dyskinesias
  - Gait imbalance, freezing

Copyright pending – do not reproduce without permission

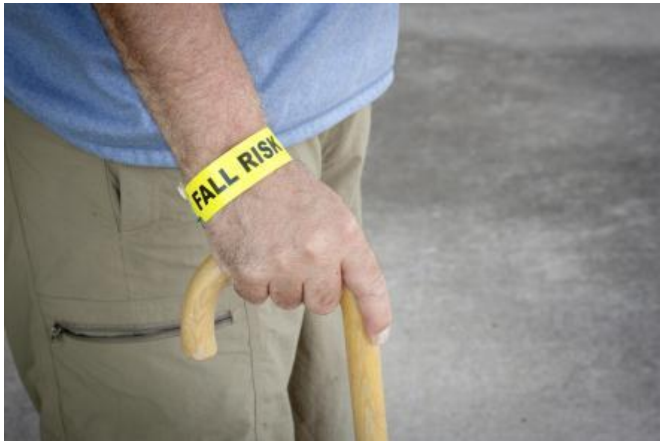

Life with PD

- Patients may be late
  - *What if leaving the house took 3 times as long?*
  - *What if driving & parking were twice as stressful?*
  - *What if you checked the map multiple times before arriving and still got lost?*
  - *What if you used the restroom twice before arriving but still felt you might have an accident as you waited for the elevator?*

Copyright pending – do not reproduce without permission

How Can We Help?

- Patients may be late
  - Greet them with a smile, “glad you were able to make it”, and benefit of the doubt
    - Traffic is a big source of anxiety for people with parkinsonism
    - Visuospatial processing (getting to a specific office, navigating maps) is challenging, can be a source of embarrassment

Copyright pending – do not reproduce without permission

Life with PD

- Patients may be slow
  - *What if the more you tried to hurry, the slower you became?*
  - *What if you were dependent on a pill every few hours to move?*
  - *What if you suddenly became frozen and unable to move without warning?*
  - *What if the more you tried to rush, the dizzier you became?*

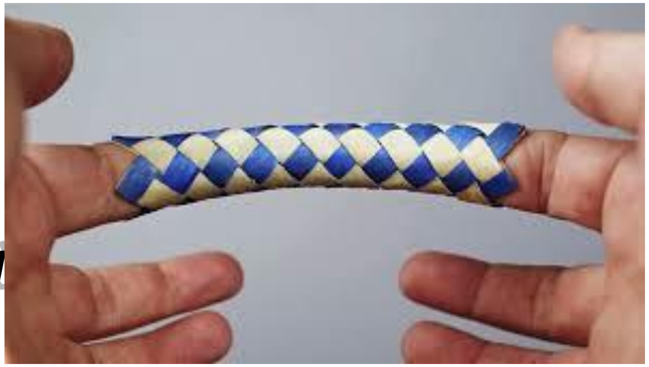

Copyright pending – do not reproduce

How Can We Help?

- Patients may be slow
  - Paradox: The more they try to rush, the *slower* things get.
  - Solution: *Patience.* Encourage them to take their time, take a breath.
    - Minimize any multitasking or distractions
  - Bradyphrenia (slow thinking/processing):
    - Allow an extra few seconds for an answer

Copyright pending – do not reproduce without permission

Life with PD

- Patients may be hard to understand
  - *What if you thought you were speaking at full volume, but no one could hear you?*
  - *What if you couldn't think of the word you wanted to say when everyone was watching?*

Copyright pending – do not reproduce without permission

How Can We Help?

- Patients may be hard to understand
  - Hypophonia: Quiet voice that gets quieter, more rushed as person speaks
  - Dysarthria: Slurred speech
  - Word-finding difficulties
  - Pressured or tangential speech

Copyright pending – do not reproduce without permission

- Ask simple, clarifying questions: “It sounds like you said….”, “Did you mean…?”

KICK-OUT PD

- *Karate Intervention to Change Kinematic OUTcomes in Parkinson's Disease*

- First of its kind pilot study of a karate intervention for people with early to middle-stage PD

- Supported by private philanthropic donation

Copyright pending – do not reproduce without permission

KICK-OUT PD

- Rationale: Research shows that…
  - Aerobic exercise has symptomatic and neuroprotective benefits in PD
  - Tai chi, yoga, and pilates have benefits for strength and balance in PD
  - Mindfulness training and meditation may be helpful for attention, anxiety, and depression in PD

Copyright pending – do not reproduce without permission

KICK-OUT PD

- Strong enthusiasm for boxing in PD, especially among people who have not exercised otherwise
  - Aerobic benefits, plus ? emotional benefit
- If aerobic exercise, group exercise, tai chi, and mindfulness are each beneficial….

…could karate for PD combine these?

…could it be even more powerful than the individual disciplines?

Copyright pending – do not reproduce without permission

KICK-OUT PD

Pre-Intervention Study Visit

- At Rush
- Basic health information; test balance, gait, attention, anxiety, depression
- Focus group to discuss past experience with martial arts and exercise, expectations

Karate Classes

- Wilmette and Park Ridge Dojos
- **10 weeks long**
- **Each participant attends 2 classes per week** at one dojo location
- 15 participants per class

Post-Intervention Study Visit

- Basic health information; test balance, gait, attention, anxiety, depression
- Focus group to discuss experience with classes, goals achieved, future directions

Copyright pending – do not reproduce without permission

KICK-OUT PD

- We are measuring changes in:
  - Balance
  - Gait
  - Flexibility
  - Attention
  - Anxiety
  - Depression
- Participant’s overall impression of change

Copyright pending – do not reproduce without permission

Questions?

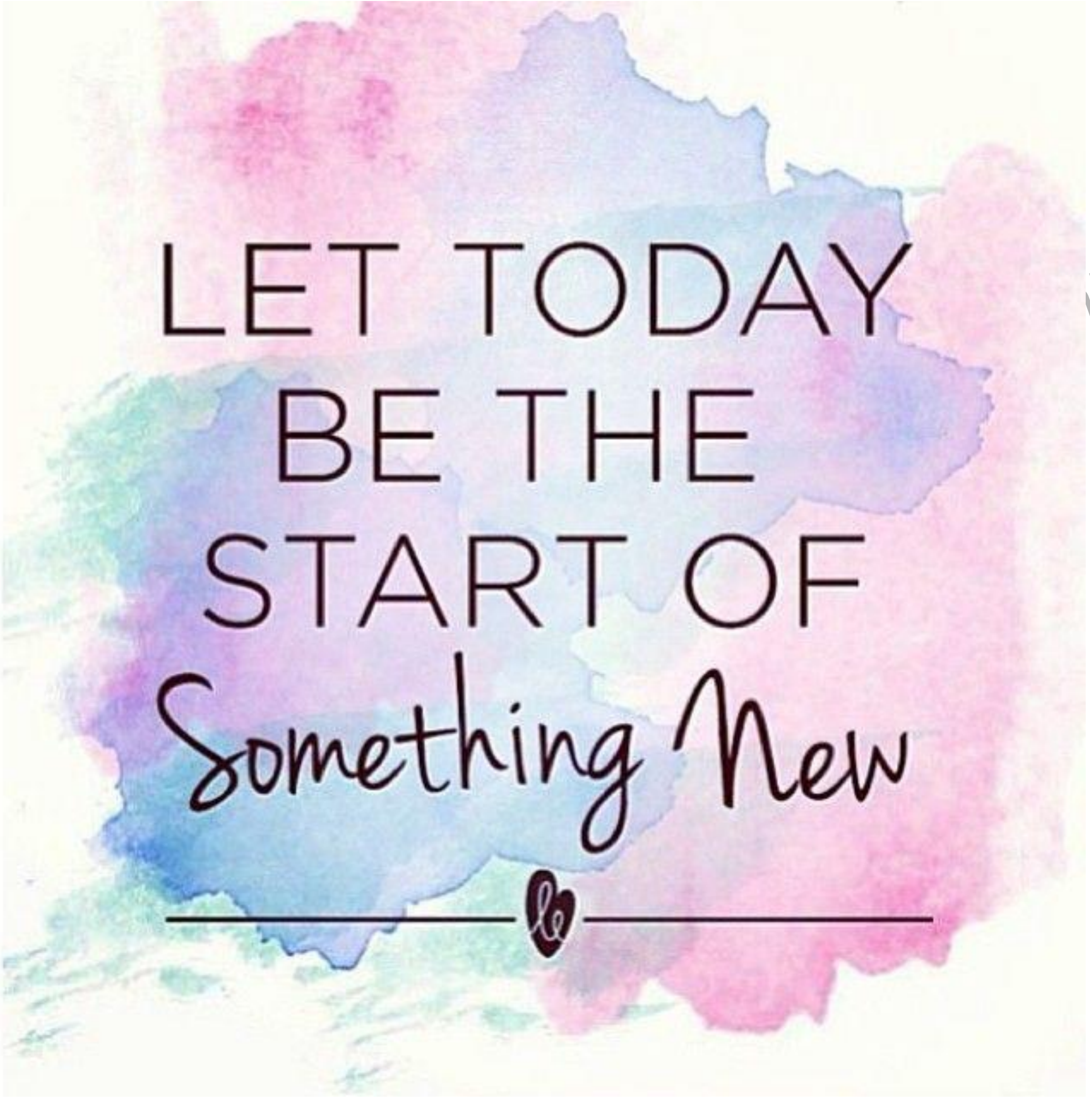

Copyrig
ission
