# Supplementary material for: KICK OUT PD: Feasibility and quality of life in the pilot karate intervention to change kinematic outcomes in Parkinson’s Disease
Source: PLoS One. 2020 Sep 9;15(9):e0237777. doi: 10.1371/journal.pone.0237777 (PMC7480843; doi:10.1371/journal.pone.0237777)
Supplement: S3 Appendix — (DOCX) [file pone.0237777.s003.docx]

**S3 Appendix**

**KICK-OUT PD**

An intervention description for the pilot study of KICK-OUT PD (Karate Intervention to Change Kinematic Outcomes in Parkinson’s Disease) (Item 1) including all TIDieR items

**Rationale (Item 2)**

The rationale for offering this community-based karate class to individuals with early-to-middle stage PD is described in the Introduction section of this article.

**Materials (Item 3)**

The karate instructors were provided with a training program to provide information on the clinical features of Parkinson’s disease, the importance of exercise, strategies for best supporting people with PD, and information on the study. The training was presented as a series of Power Point slides that are available as S2 appendix.

**Procedures (Item 4)**

All participants attended karate classes in the community where specially trained karate instructors taught the curriculum, which can be accessed in full in S1 appendix. The curriculum progressed over the course of the ten-weeks of classes as the participants advanced in their learning and skill. Each session varied and included components of warming up, stationary basics, stance work, strikes from fighting stance, kata, agility and coordination exercises, conditioning, practice falling, and cool-down/meditation. While mindfulness and breathing exercises are a specific part of each class, there is also an emphasis on mindful body and spatial awareness throughout the entire practice of karate. All classes are non-contact, so strikes and kicks were directed in the air or at a punching bag or pad held by an instructor. All classes took place on the padded floor of the karate studio.

- Warm-ups included marching/jogging in place, kick-backs, knee-ups, joint mobility, and stretching
- Stationary basics consisted of holding a stance and performing specific arm movements, including blocks and strikes with focus and power.
- Stance work consisted of holding a specific stance and moving across the floor, incorporating strikes and blocks
- Strikes from fighting stance consisted of jabs and punches incorporated with stance work, both in the air and directed at a punching bag, mitt, or pad held by an instructor.
- Kata was incorporated in later classes and is a traditional series of choreographed movements that incorporates stances, strikes, blocks, and moves across the floor
- Agility and conditioning exercises consisted of footwork using an agility ladder, small hurdles (6 inches off the ground), and core muscle strengthening.
- Practice falling included techniques to safely catch oneself and control a fall.
- Cooldown included static stretches, focus on deep breathing and other mindfulness techniques including visualizations and bringing awareness to ones’ body.
- The details of each component can be accessed in S1 appendix.

**Providers (Item 5)**

The community-based karate classes were all taught by karate instructors with at least one year of professional karate teaching experience with adults, and who had achieved a black belt in karate themselves. All instructors completed PD-specific training with the principal investigator, as described above. Two or three instructors, plus at least one study team member, were present at every class.

**How (Item 6)**

The karate classes were taught in-person in a group class setting. Each class consisted of only participants in this study.

**Where (Item 7)**

The classes took place at two community-based karate studios (dojos) in the Chicago suburbs.

**When and How Much (Item 8)**

Each class session was one hour long and the study consisted of two classes per week for ten weeks. There were two locations running the curriculum simultaneously, so the participants could make up a missed class at the other location if necessary.

**Tailoring (Item 9)**

The curriculum was taught in a group setting and adjustments were made based on individual participant’s needs on an ongoing basis in order to ensure safety. If a participant was feeling increased imbalance, they would be encouraged to widen their stance and focus on arm work.

**Modifications (Item 10)**

The curriculum was expected to progress and advance over the course of the study as participants learned skills and gained confidence. There were no modifications to the overall study design.

**Planned: Adherence and fidelity (Item 11)**

Adherence to class participation was tracked through attendance. Instructors took attendance at every class and these records were shared with the study team. Withdrawal from study participation was monitored based on >2 missed classes with subsequent phone call follow-up from the study staff, or direct contact from participants if they needed to leave the study for any reason.

**Actual: Adherence and fidelity (Item 12)**

Withdrawal from the study was 4/19 participants (21.05%). Mean attendance among the 15 participants completing the study was 86.7% of all classes.

**The TIDieR (Template for Intervention Description and Replication) Checklist*:**

Information to include when describing an intervention and the location of the information

| **Item number** | **Item** | **Where located **** | |
| --- | --- | --- | --- |
|  |  | Primary paper  (page or appendix  number) | Other ^†^ (details) |
|  | **BRIEF NAME** |  |  |
| **1.** | Provide the name or a phrase that describes the intervention. | ___5_________ | ______________ |
|  | **WHY** |  |  |
| **2.** | Describe any rationale, theory, or goal of the elements essential to the intervention. | ___3-5_________ | _____________ |
|  | **WHAT** |  |  |
| **3.** | Materials: Describe any physical or informational materials used in the intervention, including those provided to participants or used in intervention delivery or in training of intervention providers. Provide information on where the materials can be accessed (e.g. online appendix, URL). | __13__________ | ____S2 Appendix_________ |
| **4.** | Procedures: Describe each of the procedures, activities, and/or processes used in the intervention, including any enabling or support activities. | ___12_________ | __S1 appendix___________ |
|  | **WHO PROVIDED** |  |  |
| **5.** | For each category of intervention provider (e.g. psychologist, nursing assistant), describe their expertise, background and any specific training given. | __13__________ | _____________ |
|  | **HOW** |  |  |
| **6.** | Describe the modes of delivery (e.g. face-to-face or by some other mechanism, such as internet or telephone) of the intervention and whether it was provided individually or in a group. | ___12-13_________ | _____________ |
|  | **WHERE** |  |  |
| **7.** | Describe the type(s) of location(s) where the intervention occurred, including any necessary infrastructure or relevant features. | ___12-13__________ | _____________ |
|  | **WHEN and HOW MUCH** |  |  |
| **8.** | Describe the number of times the intervention was delivered and over what period of time including the number of sessions, their schedule, and their duration, intensity or dose. | __12-13___________ | _____________ |
|  | **TAILORING** |  |  |
| **9.** | If the intervention was planned to be personalised, titrated or adapted, then describe what, why, when, and how. | ___13__________ | _____________ |
|  | **MODIFICATIONS** |  |  |
| **10.^ǂ^** | If the intervention was modified during the course of the study, describe the changes (what, why, when, and how). | __N/A___________ | _____________ |
|  | **HOW WELL** |  |  |
| **11.** | Planned: If intervention adherence or fidelity was assessed, describe how and by whom, and if any strategies were used to maintain or improve fidelity, describe them. | ___13__________ | _____________ |
| **12.^ǂ^** | Actual: If intervention adherence or fidelity was assessed, describe the extent to which the intervention was delivered as planned. | __17___________ | _____________ |

** **Authors** - use N/A if an item is not applicable for the intervention being described. **Reviewers** – use ‘?’ if information about the element is not reported/not sufficiently reported.

† If the information is not provided in the primary paper, give details of where this information is available. This may include locations such as a published protocol or other published papers (provide citation details) or a website (provide the URL).

ǂ If completing the TIDieR checklist for a protocol, these items are not relevant to the protocol and cannot be described until the study is complete.

* We strongly recommend using this checklist in conjunction with the TIDieR guide (see *BMJ* 2014;348:g1687) which contains an explanation and elaboration for each item.

* The focus of TIDieR is on reporting details of the intervention elements (and where relevant, comparison elements) of a study. Other elements and methodological features of studies are covered by other reporting statements and checklists and have not been duplicated as part of the TIDieR checklist. When a **randomised trial** is being reported, the TIDieR checklist should be used in conjunction with the CONSORT statement (see [www.consort-statement.org](http://www.consort-statement.org)) as an extension of **Item 5 of the CONSORT 2010 Statement.** When a **clinical trial** **protocol** is being reported, the TIDieR checklist should be used in conjunction with the SPIRIT statement as an extension of **Item 11 of the SPIRIT 2013 Statement** (see [www.spirit-statement.org](http://www.spirit-statement.org)). For alternate study designs, TIDieR can be used in conjunction with the appropriate checklist for that study design (see [www.equator-network.org](http://www.equator-network.org)).
